# CRISPR Technology Acts as a Dual-Purpose Tool in Pig Breeding: Enhancing Both Agricultural Productivity and Biomedical Applications

**DOI:** 10.3390/biom14111409

**Published:** 2024-11-05

**Authors:** Bo Fu, Hong Ma, Xiupeng Huo, Ying Zhu, Di Liu

**Affiliations:** 1 Institute of Animal Husbandry, Heilongjiang Academy of Agricultural Sciences, Harbin 150086, China; fubo@haas.cn (B.F.); hongma@haas.cn (H.M.); 2 Key Laboratory of Combining Farming and Animal Husbandry, Ministry of Agriculture and Rural Affairs, Harbin 150086, China; 3 College of Wildlife and Protected Area, Northeast Forestry University, Harbin 150040, China; h2218794971@163.com; 4 The Breeding Center of Felid of Hengdao He Zi (Heilongjiang), Harbin 150028, China; wwt@neau.edu.cn

**Keywords:** pig, gene editing, CRISPR/Cas9, recombination, transgenic technology

## Abstract

Pigs have long been integral to human society for their roles in agriculture and medicine. Consequently, there is an urgent need for genetic improvement of pigs to meet human dual needs for medicine and food. In agriculture, gene editing can improve productivity traits, such as growth rate and disease resistance, which could lower farming costs and benefit consumers through enhanced meat quality. In biomedical research, gene-edited pigs offer invaluable resources as disease models and in xenotransplantation, providing organs compatible with human physiology. Currently, with CRISPR technology, especially the CRISPR/Cas9 system emerging as a transformative force in modern genetics, pigs are not only sources of sustenance but also cornerstones of biomedical innovation. This review aims to summarize the applications of CRISPR/Cas9 technology in developing pigs that serve dual roles in agriculture and biomedical applications. Compared to ZFNs and TALENs, the CRISPR/Cas9 system offers several advantages, including higher efficiency, greater specificity, ease of design and implementation, and the capability to target multiple genes simultaneously, significantly streamlining the process of genetic modifications in complex genomes. Therefore, CRISPR technology supports the enhancement of traits beneficial for agricultural productivity and facilitates applications in medicine. Furthermore, we must acknowledge the inherent deficiencies and technical challenges of the CRISPR/Cas9 technology while also anticipating emerging technologies poised to surpass CRISPR/Cas9 as the next milestones in gene editing. We hypothesize that with the continuous advancements in gene editing technologies and successful integration of traits beneficial to both agricultural productivity and medical applications, the goal of developing dual-purpose pigs for both agricultural and medical use can ultimately be achieved.

## 1. Introduction

Pigs have been essential to human society for thousands of years [1], providing a critical source of food, agricultural productivity, and, more recently, medical advancements [2,3]. On the one hand, pigs, as an essential source of meat, account for approximately 35% of all global meat production, making it a staple food in many cultures [2]; consequently, genetic improvements are necessary to enhance the nutritional value, optimize meat quality, improve taste, and increase disease resistance in pigs [4,5,6,7]. On the other hand, because porcine organ sizes are comparable to those of humans and pigs share many physiological similarities with humans, genetic modifications should be made to avoid the immune system’s rejection of porcine organs or to construct models for studying human diseases [3,8,9,10,11]. In other words, the ultimate goal of genetically modifying pigs is to achieve dual use for both medicine and food.

Although the initial applications of gene editing technologies were primarily focused on plants and basic research fields, in recent years, gene editing technologies have gradually become mainstream in livestock, especially in pig breeding and biomedical models, which provided precise and efficient ways to manipulate genomic sequences and made it possible to enhance desirable traits and improve overall livestock production. Meganucleases, including I-SceI, were identified and initially characterized in the late 1980s, and the application of these enzymes in mammalian cell genome engineering was most prominent during the period from 1990 to 2000, with the discovery that nucleases can be engineered to generate site-specific DNA double-strand breaks (DSBs), thereby enhancing homologous recombination [12,13,14]. Despite being revolutionary, gene editing technology based on homologous recombination was inefficient and cumbersome, limiting its applications [15]. Zinc finger nucleases (ZFNs) developed in the 1990s and transcription activator-like effector nucleases (TALENs) in the early 2000s represented significant advances in gene editing by enabling site-specific DNA breaks [16,17], while their complexity, cost, and design challenges limited broad adoption [18]. In 2012, Martin et al. utilized a dual RNA structure to induce site-specific DNA cleavage, then demonstrated that Cas9 is an RNA-guided DNA endonuclease in bacterial immunity. The significant theoretical discovery of this study is the programmability of Cas9, which revolutionized gene editing with its precision, efficiency, and cost-effectiveness, facilitating applications across various domains [19]. The impact of the CRISPR/Cas9 system is so profound that Emmanuelle Charpentier and Jennifer Doudna, the pioneers of this technology, were awarded the Nobel Prize in Chemistry in 2020. They developed CRISPR-Cas9 as a precise genome-editing tool that allows for targeted DNA modifications in living cells. Their innovation included designing a simplified, single-guide RNA (sgRNA) system, significantly advancing genetic engineering applications. The CRISPR/Cas9 system’s technological advantage lies in its simplicity and versatility, as it can be easily programmed using guide RNA to target specific DNA sequences, enabling precise genome editing [19]. Currently, with these technological advantages, the CRISPR/Cas9 system has been widely applied in various fields of pig breeding, including enhancing growth traits, modifying meat quality, improving disease resistance, avoiding the immune system’s rejection of porcine organs, constructing disease models, and so on.

This review primarily discusses the advancements and significant achievements of the CRISPR/Cas9 technology in pig applications, while also summarizing and analyzing some inherent limitations of the technology. At the same time, it should be noted that some novel technologies in the field of gene editing, such as bridge RNA-guided recombination systems, are demonstrating unique technical advantages [20]. We hypothesize that, with the continuous advancements in synthetic biology, algorithmic improvements, and precision tools for gene editing, the technical limitations of the CRISPR/Cas9 system will gradually be overcome. In the near future, it is highly likely that new gene-editing technologies will emerge, surpassing CRISPR/Cas9 and becoming the next milestone in the evolution of gene editing.

## 2. Theoretical Framework of the CRISPR/Cas9 System

The CRISPR/Cas9 system is derived from a bacterial adaptive immune system, which evolved to protect against viral infections by recognizing and cleaving foreign DNA [19]. The core mechanism involves two key components: the CRISPR-associated Cas9 protein that cleaves DNA and a guide RNA (gRNA) that directs Cas9 to the target sequence through complementary base pairing [21]. The gRNA is a fusion of CRISPR RNA (crRNA) and trans-activating crRNA (tracrRNA), which together guide Cas9 to the target DNA sequence [22]. The protospacer adjacent motif (PAM) sequence is typically a short, conserved nucleotide motif located immediately adjacent to the target DNA sequence recognized by the gRNA. It serves as a critical recognition signal for Cas9, ensuring that the protein only binds to DNA sequences near a PAM, thus avoiding off-target effects in the genome [19]. Therefore, the specificity of the CRISPR/Cas9 system is primarily determined by the gRNA’s spacer sequence and the PAM [23]. The recognition of PAM by Cas9 occurs before the gRNA-DNA hybridization, where the enzyme undergoes a conformational change that increases its affinity for the gRNA-DNA complex only if a PAM sequence is present [24]. The requirement for PAM sequences limits the sites that Cas9 can target, thereby providing an additional layer of specificity that complements the guide RNA recognition of the target DNA [25]. Furthermore, different Cas9 variants recognize distinct PAM sequences, which expands the versatility of the CRISPR/Cas9 system across various organisms and target sites [26]. Upon recognizing the target sequence, Cas9 introduces a double-strand break (DSB) at the specific genomic location [22]. This DSB is repaired by one of two cellular repair pathways: non-homologous end joining (NHEJ) or homology-directed repair (HDR). NHEJ, which often results in insertions or deletions, leads to gene disruption, and HDR can introduce specific changes if a repair template is provided [19,27]. The CRISPR/Cas9 technology has demonstrated numerous advantages over previous genome editing technologies such as ZFNs and TALENs. One of the primary advantages of the CRISPR/Cas9 system is its simplicity and ease of design, as it only requires a guide RNA (gRNA) to direct the Cas9 nuclease to the target DNA sequence, compared to the complex protein engineering needed for ZFNs and TALENs [28]. This simplicity also makes the CRISPR/Cas9 system more cost-effective and faster to implement. The CRISPR/Cas9 system is also more versatile in terms of applications. It can be used not only for gene knockout but also for gene knock-in, gene regulation, and epigenetic modifications [29]. Although peptide-directed methods primarily rely on the specific binding of proteins, offering greater target specificity and naturally lower error rates compared to single-guide RNA methods, the slight increase in off-target effects with sgRNAs is acceptable given the ease of use of CRISPR’s sgRNA system [30]. Currently, based on the above-mentioned technical advantages, CRISPR/Cas systems have become the mainstream gene editing method in livestock breeding.

## 3. Methods for Generation of Genetic Modified Pigs Using the CRISPR/Cas9 System

Currently, CRISPR/Cas9 systems are used to modify the genomes of pigs via various methods, including somatic cell nuclear transfer (SCNT), microinjection, electroporation, and lipofection. SCNT entails the selection of somatic cells, typically fetal fibroblasts that have undergone specific genetic modifications, followed by the nuclear transfer of these altered cells in the cloning procedure. In this process, Cas9 nucleases facilitate the generation of mutations via non-homologous end joining (NHEJ) or homology-directed repair (HDR) in donor cells in vitro, utilizing selection strategies to enrich for cells harboring the targeted mutations. Subsequent to the meticulous selection of donor cells post-gene editing, the delivered piglets carry the desired genotypes. In the field of genetic engineering, knock-out refers to the deletion or disabling of a specific gene to eliminate its function, while knock-in involves inserting an external gene at a specific locus to introduce new or modified gene expression. The CRISPR/Cas9 system enables the editing of genes and the insertion of exogenous genes, resulting in gene knockout pigs, even knock-in pigs, through the use of the SCNT technique [31,32,33,34].

An alternative to the SCNT method involves direct gene editing via the direct introduction of gene editors during embryogenesis. Microinjection of gene editors into zygotes/embryos is a straightforward technique that simplifies the genetic modification of fertilized zygotes or embryos. It is well known that the pronuclear injection process requires that the pronuclei be visible. Thus, due to the significant lipid content in porcine zygotes, centrifugation was needed to render the pronuclei visible for successful pronuclear injection during the zygote stage. Nonetheless, gene editors are typically equipped with nuclear localization signals, obviating the need for centrifugation and the precise positioning of the glass needle towards the pronuclei. Furthermore, microinjection facilitates the incorporation of large molecules, thereby allowing for the creation of microinjection-mediated gene knock-in. In addition, compared with SCNT-generated embryos, the viability of the zygotes/embryos manipulated through microinjection was higher, and the resultant litters from these embryos are typically larger than those derived from SCNT-generated embryos [35]. Currently, pigs that have undergone gene editing via cytoplasmic microinjection have been successfully developed by introducing gene editors early in their developmental stages [36,37,38,39].

Delivery of the CRISPR/Cas9 system via electroporation is another alternative to SCNT. Electroporation-mediated gene editing represents a method devoid of micromanipulation, allowing for the preparation of large quantities of gene-edited embryos through the introduction of gene editors into zygotes. Electroporation-mediated gene editing requires no complex micromanipulation procedures such as enucleation and nuclear injection, along with the associated costly micromanipulation equipment. Whereas the introduction of transgenes for knock-in applications via electroporation also encounters challenges in pigs. It is widely acknowledged that in vitro-fertilized porcine zygotes/embryos exhibit sensitivity to electrical fields, and high voltages prove detrimental, a contrast to the tolerance observed in mice [40,41], and the efficiency of molecular uptake into cells through electroporation correlates with the field strength, pulse duration, and the number of pulses applied. Consequently, a viable knock-in system for introducing large transgenes via electroporation has not yet been established. Currently, electroporation has been effectively applied to gene editing in porcine zygotes, primarily achieving gene knockout [40,42,43].

**Figure 1:**
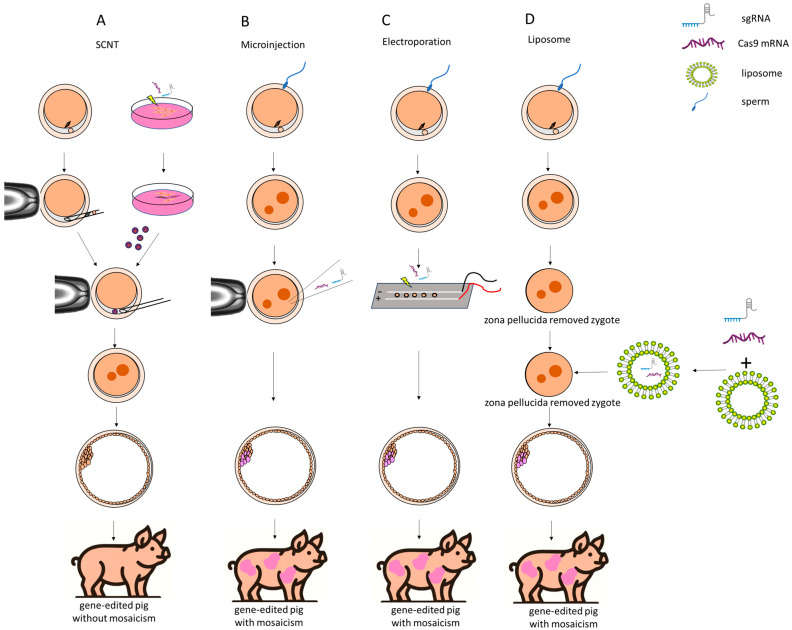
Four methods for creating gene-edited pigs using CRISPR/Cas9 technology. (**A**) Somatic cell nuclear transfer. A donor cell, which has been edited with CRISPR/Cas9, is transferred into an enucleated porcine oocyte. The edited nucleus integrates into the oocyte, ultimately producing gene-edited pigs without mosaicism. (**B**) Microinjection. CRISPR/Cas9 components, such as sgRNA and Cas9 mRNA, are directly injected into a fertilized zygote. This leads to a gene-edited pig, but there is a possibility of mosaicism. (**C**) Electroporation. The CRISPR/Cas9 components are delivered into the zygote using an electric field. The zygote is placed between electrodes, and an electric pulse is applied to create pores in the membrane, allowing the CRISPR/Cas9 complex to enter the cells. Similarly to microinjection, this method may also produce gene-edited pigs with mosaicism. (**D**) Liposome. The CRISPR/Cas9 components are encapsulated in liposomes, which merge with the zygote after the zona pellucida is removed. Thus, the liposomes help deliver the CRISPR components into the zygote for gene editing. Liposome-mediated delivery is simpler but may also result in mosaicism.

Aside from electroporation, liposome-mediated gene transfer, an equipment-free method, has also become an alternative method for establishing a gene targeting framework. It is widely recognized that gene transfer techniques, including SCNT, microinjection, and electroporation, necessitate specialized and costly equipment. Whereas Hirata et al. propose an alternative method using lipofection to introduce CRISPR/Cas9 components into cells, focusing on zona pellucida-free oocytes and embryos [44]. In more detail, Hirata et al. employed lipofection using Lipofectamine 2000 (Thermo Fisher Scientific, Waltham, MA, USA) to deliver Cas9 protein and guide RNA targeting two specific genes, *PDX1* and *GGTA1*, then sanger sequencing and TIDE analysis were used to determine gene editing efficiency. Results have shown that lipofection successfully introduced the CRISPR/Cas9 system into the embryos. However, mosaic mutations were observed at various stages of embryonic development, and the overall efficiency of gene editing was lower compared to traditional methods such as microinjection or electroporation [44]. Despite these limitations, the study’s practical value lies in the fact that lipofection can be used as an equipment-free method for CRISPR/Cas9 delivery in porcine embryos, making gene editing more accessible to laboratories lacking specialized equipment. Its innovation comes from applying lipofection, a technique traditionally used in cell cultures, to early-stage embryos for genome editing. This could pave the way for more cost-effective methods of producing genetically modified pigs, although further improvements are needed for its widespread application. In this review, we compared four methods for the generation of genetically modified pigs using the CRISPR/Cas9 system, as shown in Table 1. The construction methods for the CRISPR/Cas9 system-guided four types of gene-edited pigs mentioned above are shown in Figure 1.

A significant advantage of SCNT over direct embryo injection lies in the predictable genotype of the founder pigs. In detail, the SCNT technique enables the production of genetically identical clones by transferring an edited somatic cell nucleus into an enucleated oocyte, thus avoiding mosaicism and ensuring editing accuracy. Additionally, SCNT allows for genetic analysis of donor cells before implantation, verifying that the edits meet experimental expectations, which enhances reliability and efficiency. Conversely, pigs produced through embryo injection typically exhibit mosaic genotypes, characterized by various modification types across different cells, and multiple breeding cycles are often required to produce pigs that are homozygously modified and possess identical genotypes. In certain cases, the chimeric founders retain intact wild-type (WT) germ cells, thereby precluding the generation of genetically modified offspring. However, it should be kept in mind that SCNT of genome-edited cells may be compromised by impaired embryonic development, likely caused by off-target effects, and genome-edited somatic cells typically exhibit lower cloning efficiency compared to wild-type (WT) or randomly integrated transgenic cells. The process of SCNT is technically demanding, involving the enucleation of oocytes and the insertion of a donor nucleus. This complexity requires highly skilled operators and can lead to inconsistencies in results due to variations in technique. In addition, offspring generated from genetically modified reconstructed embryos frequently exhibit abnormalities, including birth defects, miscarriages, and early postnatal mortality, which are common complications associated with SCNT. Due to the heightened sensitivity of in vitro-fertilized porcine zygotes to electrical fields compared to in vivo-derived mouse embryos, there are restrictions on the size of molecules that can be introduced into these zygotes and embryos [40,41]; therefore, electroporation is suitable only for gene knockout and not for gene knockin [40,42,43]. In addition, with an increase in the number of genes targeted simultaneously, there is a corresponding rise in the risk of incomplete gene knockouts, leading to a higher occurrence of mosaicism; thus, when it comes to multi-gene editing, SCNT is predominantly employed as the principal technique [45]. Taken together, the method of gene editing should be chosen based on the experimental objectives.

## 4. Application of the CRISPR/Cas9 System in Gene Editing Pigs

### 4.1. CRISPR/Cas9 System in Gene Editing Pigs in Agriculture

#### 4.1.1. Improving the Productive Performance of Pigs

Improving muscle quality and reducing fat tissue are objectives in commercial pig farming. The myostatin (*MSTN*) gene is a negative regulator of skeletal muscle growth and development, and the knockout of this gene results in animals exhibiting a double-muscled phenotype with increased muscle mass. Therefore, MSTN, which acts as an inhibitor of skeletal muscle development, is a research target for increasing muscle mass in livestock. The knockout of the *MSTN* gene in pigs via the CRISPR/Cas9 system not only significantly increases lean meat production [5,46], but also substantially raises the levels of polyunsaturated fatty acids [47]. The follistatin (FST) molecule, known for its MSTN-inhibiting properties, has potential to significantly increase muscle mass. Utilizing an HMEJ strategy enhances the efficiency of gene knock-in by integrating a specially designed FSI-I-I construct into the *MSTN* gene. In detail, experiments in this paper showed that HMEJ increased knockin rates at the *pRosa26* and *pACTB* loci by approximately threefold compared to HDR, with knockin efficiencies of 20.83% ± 0.7881% and 15.40% ± 0.3606%, respectively, in porcine fetal fibroblasts, compared to HDR’s 7.69% ± 0.3331% and 4.98% ± 0.2961%. This method is validated through histological analysis and molecular signaling pathway investigations, indicating a robust increase in muscle mass in genetically modified pigs [48]. It should be kept in mind that newborn homozygous MSTN knockout piglets of the Landrace breed exhibit abnormal limb development, leading to severely impaired motor function and rapid postnatal death. *FBXO40* is a muscle-specific gene previously studied in mice, where it regulates the IGF1/Akt pathway by targeting IRS1 for ubiquitination, thereby also controlling muscle mass. Zou et al. used CRISPR/Cas9 combined with SCNT to create *FBXO40* knockout pigs, finding that the knockout stimulated the IGF1/Akt pathway, leading to a 4% increase in muscle mass without pathological effects in major organs. This study offers a safer alternative to *MSTN* knockout with innovative use of CRISPR/Cas9 [31]. The insulin-like growth factor 2 (*IGF2*) gene also regulates muscle mass, and precise editing of this gene can significantly increase pork muscle yield. By modifying the ZBED6 binding site in intron 3 of the *IGF2* gene using CRISPR/Cas9 genome editing technology, muscle mass in native Chinese pig breeds such as Chinese Bama pigs and Liang Guang Small Spotted pigs can be greatly increased [6,49]. In detail, editing the ZBED6 binding site in the *IGF2* gene using CRISPR/Cas9 significantly upregulated IGF2 expression. Specifically, at 338 days, the edited pigs weighed 32% more than the non-edited wild-type control pigs. Editing the *IGF2* intron 3 site in Bama pigs significantly enhanced muscle growth and lean meat percentage. In detail, edited F1 Bama pigs weighed an average of 34.58% more than the control group (WT) at six months, with lean meat weight having increased by 33.35%. Additionally, muscle tissue section analysis indicated that this muscle growth effect resulted from muscle fiber hypertrophy rather than an increase in the number of fibers [6,49]. In terms of meat quality, the pivotal role of n-3 polyunsaturated fatty acids (*PUFAs*) in human health is well-established, with deficits linked to common diseases. The *fat-1* gene from *Caenorhabditis elegans* can address these deficits by converting n-6 to n-3 PUFAs. Previous studies have successfully demonstrated the health benefits in transgenic models but faced challenges with gene expression stability and biosafety due to random integration techniques. Li et al. utilized the CRISPR/Cas9 system for site-specific integration of the *fat-1* gene into the *Rosa26* locus, achieving controlled, stable expression. Thus, this transgenic model offers a scalable and safer approach to enhancing the nutritional value of pork, which could lead to improved human health outcomes by increasing the dietary intake of n-3 PUFAs [4]. You et al. even created a double-gene knock-in pig by simultaneously inserting the *fat-1* and *IGF-1* genes into the Rosa26 locus, which simultaneously enhanced both muscle mass and meat quality in pigs [50].

Pigs lack a functional *UCP1* gene, leading to poor thermoregulation and a propensity for fat accumulation, which are significant issues in pig farming. The absence of the *UCP1* gene in pigs is a well-documented evolutionary trait that affects their susceptibility to cold and influences fat deposition. Previous research has shown the role of *UCP1* in thermogenesis in other mammals, which indicated its potential benefits in pigs. The CRISPR/Cas9 system may be used to reintroduce the *UCP1* gene to address these challenges. Recently, Zheng et al. successfully introduced the mouse *UCP1* gene into the pig genome. The genetically modified pigs exhibited improved thermoregulation and decreased fat deposition without affecting their overall energy expenditure or physical activity levels [51]. The findings suggest that introducing *UCP1* into pigs can enhance their welfare and economic value by improving cold tolerance and increasing lean meat production without compromising their normal physiological functions. In detail, Zheng et al. successfully introduced the mouse *UCP1* gene into the pig genome at the endogenous UCP1 locus using a CRISPR/Cas9-mediated homologous recombination-independent approach.

It should be kept in mind that the high costs and technical demands of producing genetically modified pigs, especially with CRISPR/Cas9, hinder their commercial scalability and widespread adoption. Genetically modified organisms (GMOs), especially those intended for food consumption, face rigorous regulatory scrutiny. Many countries impose strict requirements for the approval of GMO animals, which can involve extensive testing to assess environmental impact, safety for human consumption, and animal welfare concerns. Taken together, all these factors limit the commercialization of genetically modified pigs.

#### 4.1.2. Improving the Viral Resistance of Pigs

Porcine Reproductive and Respiratory Syndrome (PRRS) is an economically significant disease in swine, and the *CD163* gene is a receptor for the PRRS virus. Whitworth et al. employed the CRISPR/Cas9 system to create *CD163* knockout pigs and tested these animals’ susceptibility to the PRRSV, demonstrating that these gene-edited pigs did not exhibit signs of infection, viremia, or associated pathology, which indicated that *CD163* gene editing offers a viable strategy to enhance resistance to PRRS in swine, potentially reducing significant economic losses in the swine industry [7].

It is well known that amino peptidase N (ANPEP) acts as a receptor for coronavirus infections, specifically transmissible gastroenteritis virus (TGEV) and porcine epidemic diarrhea virus (PEDV), which cause significant morbidity in neonatal pigs. The ANPEP knockout (KO) in pigs revealed crucial insights into viral resistance, particularly showing that while ANPEP is essential for TGEV infection, ANPEP KO pigs remain resistant to this virus. However, these KO pigs still displayed susceptibility to PEDV, suggesting alternative entry mechanisms or receptors for PEDV. This research underscores the potential of ANPEP-KO models in breeding virus-resistant livestock [52].

The production of the porcine β-defensin 2 (*PBD-2*) gene, which has antimicrobial and immunomodulatory properties, can provide immunity against a range of bacterial pathogens. Huang et al. utilize the CRISPR/Cas9 system for targeted integration at the *Rosa26* locus to ensure stable expression of PBD-2 across generations. In detail, by inserting two copies of the *pbd-2* gene linked by a T2A sequence into the *Rosa26* locus and subsequently removing the neoR marker, Huang et al. achieved marker-free, site-specific expression of PBD-2. The transgenic pigs with the site-specific knock-in of the *pbd-2* gene exhibited significantly higher infection resistance, demonstrated by the reduced survival of Actinobacillus pleuropneumoniae and Streptococcus suis in cell culture supernatants from porcine ear fibroblasts (PEFs) compared to wild-type pigs. Furthermore, immunofluorescence and immunohistochemical analyses confirmed high expression levels of the PBD-2 protein in various organs of the TG pigs. Real-time quantitative PCR (RT-qPCR) further revealed that *PBD-2* mRNA expression was significantly upregulated in tissues such as the kidney, lung, and spleen. Collectively, these results indicate that the site-specific knock-in of the *pbd-2* gene endowed the TG pigs with enhanced resistance to bacterial infections [53].

RSAD2, also known as viperin, exhibits antiviral activities against a variety of RNA and DNA viruses, making it a promising candidate for developing antiviral strategies in livestock. Xie et al. used CRISPR/Cas9 to specifically integrate the *pRSAD2* gene into the porcine *ROSA26* locus in PK-15 cells and porcine fetal fibroblasts, then the generated *pRSAD2* knock-in cells and subsequently cloned pigs exhibited enhanced resistance to classical swine fever virus (CSFV) and pseudorabies virus (PRV), which may offer a sustainable approach to managing viral diseases in pigs and reduce economic losses significantly [54].

### 4.2. The CRISPR/Cas9 System in Gene Editing Pigs in Medical Field

#### 4.2.1. Improving Generation of Genetically Modified Disease Models

Pigs have become increasingly valuable as models for human diseases, and one of the most compelling reasons for using pigs as disease models is their physiological resemblance to humans. For instance, many organ systems in pigs, including the cardiovascular, respiratory, digestive, and immune systems, share similarities with those of humans in terms of size, structure, and function. Therefore, compared to smaller animals like mice or rats, pigs offer a more relevant system for understanding complex human conditions. *RUNX3* is recognized as a tumor suppressor gene, particularly in gastrointestinal cancers. Previous research has demonstrated inconsistencies in the expression of RUNX3 in cancerous tissues and its regulatory mechanisms. Recently, Lee et al. employed CRISPR/Cas9 to specifically target and knockout the *RUNX3* gene in porcine fetal fibroblasts, which were then used to create viable pig models via somatic cell nuclear transfer. The *RUNX3* knockout pig model exhibited its role in cancer mechanisms by demonstrating that the absence of the tumor suppressor gene *RUNX3* could lead to early gastric tumorigenesis, aligning with its known function in suppressing cancerous developments in human gastric cells. The successful generation of *RUNX3* knockout pigs exhibited a lack of RUNX3 expression, making them potentially useful models for human cancer studies, which enhanced the understanding of cancer mechanisms and the development of new treatments [32].

The *PDX1* gene is essential for pancreatic development and has implications in diabetes mellitus, making its study in pig models relevant due to their physiological similarities to humans. Tanihara et al. utilized the CRISPR/Cas9 system and a novel electroporation method called GEEP (gene editing by electroporation of Cas9 protein) to target the *PDX1* gene in pig zygotes, resulting in the birth of viable piglets with targeted modifications. These piglets exhibited phenotypes relevant to diabetes research, such as disrupted pancreatic development and variable glucose levels, reflecting the potential usefulness of these models in diabetes [55].

Hemophilia B, an X-linked disorder, impacts blood coagulation and leads to severe bleeding, especially in joints, which is caused by *Factor IX* gene defects. Previous research using a dog model was discussed, which facilitated an understanding of hemophilia’s pathophysiology, but dog models had limitations. While dog models have been instrumental in understanding certain genetic diseases, they have limitations due to differences in anatomy, immune response, and metabolic processes compared to humans. For example, dogs exhibit distinct responses in clotting factor mechanisms and immune modulation, leading to variations in disease presentation that are less analogous to human conditions. Pigs, in contrast, offer anatomical and physiological similarities to humans, particularly in organ size, metabolic rates, and disease progression. This makes them valuable for studying conditions such as hemophilia B, where accurate modeling of blood volume and coagulation pathways is critical [3].By employing the CRISPR/Cas9 system, Chen et al. created a porcine model by knocking in the human *Factor IX* gene into pigs, which allowed for the detailed study of bleeding patterns and therapeutic gene integration, showing partial success in reducing bleeding tendencies [56].

Huntington’s disease is caused by an abnormal expansion of the CAG trinucleotide repeat in the *HTT* gene, where normal individuals typically have fewer than 36 repeats, while affected individuals have an expansion exceeding this threshold, often reaching over 40 repeats. Yan et al. employed CRISPR-Cas9 to incorporate an expanded CAG repeat in exon 1 of the *HTT* gene, thereby creating a pig model of HD, which is physiologically more similar to humans than rodents. The pig model exhibited age-dependent neurological symptoms and selective neurodegeneration of striatal medium spiny neurons, closely mimicking the human condition. This research significantly enhances our understanding of Huntington’s disease by successfully creating a genetically modified pig model that mimics the selective neurodegeneration seen in humans, offering a more physiologically relevant platform for therapeutic development and testing [57].

Pigs, due to their similarity to humans in size, anatomy, physiology, and drug metabolism rates, are considered promising models for human diseases. However, the xenogeneic immune rejection of human tumors in pigs is a significant hurdle. The *IL2RG* gene, critical for lymphoid development and immune response, was knocked out in minipigs using CRISPR/Cas9, resulting in immunodeficient pigs. These pigs successfully supported the growth of human melanoma cells without rejection, proving the feasibility of this model for cancer studies and drug testing. This genetically modified model can improve the efficiency and predictiveness of drug development processes, representing a substantial innovation over traditional rodent models [58].

Phenylketonuria (PKU) is a genetic disorder characterized by the deficiency of the enzyme phenylalanine hydroxylase. Previous studies have established the foundational knowledge about PKU’s natural history and its genetic underpinnings. However, the phenotypic translation to models has been inadequate, especially in capturing the neurocognitive impacts observed in humans. Through the strategic use of the CRISPR/Cas9 system to create double-stranded breaks at specific sites of the pig *PAH* gene, Koppes et al. successfully developed PAH-null pigs. These genetically modified pigs exhibited classical PKU symptoms and provided a new model to study the disease’s pathophysiology and therapeutic interventions more effectively than previous rodent models [59]. In addition, the CRISPR-Cas9 system has been used to generate other pig models such as Duchenne muscular dystrophy, Laron syndrome, and Werner syndrome pig models [60,61,62]. It is reasonable to believe that with the continuous advancements in gene editing technology, the creation of disease model pigs is expected to proliferate rapidly.

#### 4.2.2. Improving Xenotransplantation Research

The size of pig organs, such as the heart, liver, and kidneys, is more comparable to human organs than those of rodents. This similarity is particularly important for research on organ transplantation and the development of surgical procedures. However, pig-to-human xenotransplantation faces immunological challenges and a risk of viral transmission. Fortunately, CRISPR/Cas9 gene-editing technology represents a transformative advancement in the field of pig-to-human xenotransplantation, offering solutions from multiple perspectives. Firstly, this technology allows precise modifications of the porcine genome, particularly in removing or altering genes that trigger immune responses in human recipients. Furthermore, it enables the elimination of porcine endogenous retroviruses (PERVs), mitigating the risk of cross-species viral transmission. In short, the CRISPR/Cas9 system facilitates further refinement of genetic modifications, making pig organs more compatible with humans and advancing the field toward the realization of widespread clinical xenotransplantation.

Specific pig genes, when inactivated, can reduce the antigenicity of pig cells to human and non-human primate antibodies. Prior literature has identified the *GGTA1* and *CMAH* genes as primary contributors to antigens that elicit strong immune responses in xenotransplantation. To reduce human and non-human primate antibody binding to pig cells and alleviate xenotransplantation rejection issues, these specific pig genes need to be knocked out. Using CRISPR/Cas9 technology, Estrada et al. successfully knocked out the *GGTA1, CMAH,* and *b4GalNT2* genes in pigs, resulting in a significant decrease in antibody binding from both humans and non-human primates to the genetically edited pig cells [63]. Subsequently, they conducted in vitro and perfusion experiments to evaluate human platelet interactions with liver sinusoidal endothelial cells from these genetically modified pigs and revealed that pigs lacking *GGTA1* and *CMAH* genes exhibited substantially reduced platelet binding and consumption compared to wild-type controls, which indicated that dual gene silencing in pigs not only diminishes the antibody-mediated rejection risks but also significantly lessens complications like thrombocytopenia associated with xenotransplantation [64].

PERVs are naturally occurring retroviruses within the pig genome capable of infecting human cells. If these viruses transfer from pig cells to human recipients during transplantation, they could cause infections. It is particularly noteworthy that the expression of PERVs might trigger immune responses in humans, exacerbating organ rejection and affecting the survival and function of the transplanted organ. Previous approaches to minimize PERV risks included the use of RNA interference and gene editing techniques like zinc finger nucleases, which had limited success. Yang et al. designed specific guide RNAs for CRISPR/Cas9 to target and disrupt the PERV pol gene across all detected copies in the PK15 pig kidney epithelial cell line and then achieved a significant reduction in PERV infectivity, with the risk of PERV transmission to human cells decreasing by over 1000-fold. The success of the CRISPR/Cas9 system suggests that similar strategies could be applied to other zoonotic elements that pose risks in animal-to-human transplants [65]. Furthermore, using CRISPR/Cas9, Niu et al. successfully inactivated all copies of the PERV genes in a primary porcine fibroblast cell line, then used these modified cells to produce PERV-inactivated pigs through somatic cell nuclear transfer, which eliminated the risk of PERV transmission and enhanced the safety of pig organs for human use [66]. The above content has been organized into Table 2.

## 5. Challenges Faced by the CRISPR-Cas9 System

CRISPR/Cas9 technology has emerged as a revolutionary tool in gene editing; however, it faces several significant challenges. One major issue is the potential for off-target effects, where CRISPR may inadvertently edit unintended genomic locations, leading to unwanted mutations and possible adverse effects on cell function [67]. This phenomenon arises from the suboptimal binding of guide RNA to the target DNA, potentially resulting in off-target mutations and genomic instability [19]. In addition, CRISPR/Cas9 primarily functions by inducing double-strand breaks (DSBs) in the target DNA, which are repaired by non-homologous end joining (NHEJ) or homology-directed repair (HDR). NHEJ is error-prone, often leading to insertions and deletions (indels) that can cause mutations [25]; thus, this approach increases the risks associated with mutagenic repair pathways. The DSBs induced by CRISPR/Cas9 can even trigger cellular checkpoints and may lead to cytotoxic effects when the damage exceeds repair capacity, which can trigger apoptosis or senescence [68]. It should be noted that CRISPR/Cas systems are originally derived from bacterial immune defense mechanisms against viruses. When these bacterial proteins are introduced into the host body, the host’s immune system may recognize them as foreign invaders, triggering an immune response. Previous studies have shown that these immune reactions can diminish the effectiveness of gene editing and, in some cases, cause serious side effects such as immune-mediated damage to tissues [69,70]. Although CRISPR/Cas9 is widely used for a variety of genomic edits, it struggles with more complex edits such as large insertions, inversions, or precise multi-locus modifications [67]. Besides this, CRISPR/Cas9 lacks a natural mechanism for precise recombination beyond simple cuts, which limits its ability to perform targeted large-scale genomic rearrangements [25]. Therefore, there is an urgent need to develop gene editing tools for insertions, deletions, inversions, or large-scale genomic rearrangements at target sites in the genome without the limitations imposed by double-strand breaks (DSBs). Theoretically, CRISPR/Cas9 can target multiple loci; in fact, its performance declines with increased multiplexing due to higher chances of off-target effects and competing guides [30,71]. Then, further research on the CRISPR/Cas9 system still needs to focus on improving the precision and reliability of multiplexed gene editing. It is worth noting that the targeting efficiency of CRISPR components varies significantly across different genomic contexts, with heterochromatic regions exhibiting lower rates of editing. This phenomenon is attributed to the dense packing of DNA in these regions that poses physical barriers to the CRISPR machinery, thus causing less access to the Cas9 enzyme and guide RNA complex. Specifically, Mali et al. revealed that in certain cell types, such as induced pluripotent stem cells, the targeting rates could be as low as 2–4% in areas of condensed chromatin, emphasizing the challenge of achieving efficient genome editing in such environments [72]. Safe-harbor sites (such as the ROSA26 locus), particularly as alternatives to heterochromatin regions for stable gene integration, provide stable and predictable expression without disrupting endogenous gene function. Previous studies have demonstrated the effective use of the ROSA26 locus in swine for stable transgene expression, including applications in xenotransplantation and disease resistance [4].Utilizing safe-harbor sites minimizes off-target effects and mitigates risks associated with random insertion into potentially deleterious genomic regions. For instance, targeted knock-ins at these sites have shown promise in expressing genes with minimal interference from surrounding genomic elements [73].

## 6. Other Promising Alternatives to CRISPR/Cas9 Technology

Besides the CRISPR/Cas9 system, various gene editing technologies have been developed, each with comparative advantages over the CRISPR/Cas9 system. Prime editing (PE) is a cutting-edge technique that originates from the CRISPR/Cas9 system, utilizing a nickase variant of Cas9 (H840A), a reverse transcriptase from the Moloney murine leukemia virus (M-MLV RT), and a pegRNA that encompasses a spacer sequence, a scaffold, a reverse transcriptase template (RTT), and a prime binding site (PBS) sequence [74]. PE facilitates base substitutions, small insertions, and deletions without necessitating double-strand breaks (DSBs) or donor DNA [75]. In comparison to CRISPR/Cas9-mediated homology-directed repair (HDR), PE exhibits reduced off-target effects, improved safety, and greater flexibility, remaining unaffected by the stage of the cell cycle [76]. Despite these advantages, PE is notably more complex in design compared to the traditional CRISPR/Cas9 system. This complexity primarily stems from the required pegRNA (prime editing guide RNA), which not only directs the editing process but also contains additional sequences necessary for reverse transcription. In detail, the pegRNA, including a spacer, a scaffold, a RTT, and a PBS, to support the targeted base changes [74]. Additionally, the efficiency of PE decreases as the size of the desired insertion increases, primarily due to limitations in the reverse transcriptase’s ability to synthesize longer stretches of DNA from the pegRNA template.

Double-strand breaks are a common feature of traditional CRISPR/Cas9 methods, while base editing technology operates on the principle of converting one DNA base into another without inducing double-strand breaks. This system utilizes a modified Cas9 protein that is catalytically inactive (defective Cas9 or dCas9) to precisely target specific genomic locations. It recruits a deaminase enzyme that chemically alters the targeted base, allowing for direct nucleotide substitution. Base editing technology was created to address the limitations of traditional gene editing methods, like CRISPR/Cas9, especially the difficulties in making precise point mutations in genomic sequences, thus making it a more valuable tool for accurate gene editing [77].However, Kim et al. demonstrated that base editors could induce off-target deaminations in genomic DNA [78], which may lead to unwanted mutations that may disrupt gene function or cause diseases. These off-target edits highlight the need for improved specificity.

Cas12a lacks the HNH domain found in Cas9, instead using a RuvC-like domain for DNA cleavage; thus, Cas12a performs “staggered” cuts, which can be beneficial for multiplexed genome editing. Cas12a also requires a T-rich PAM sequence (5′-TTTN-3′), which can increase on-target editing efficiency due to lower chances of misreading in high GC genomes [79,80]. Covalent conjugation of Cas12a with crRNA has been shown to significantly improve its genome editing efficiency, making it more precise than the wild-type Cas12a complex [81]. In addition, Cas12a’s smaller size compared to Cas9 can facilitate easier delivery into cells [82]. Therefore, CRISPR/Cas12 technology was developed to enhance genome editing capabilities by addressing the limitations of earlier CRISPR systems, particularly in terms of efficiency, precision, and the ability to target a broader range of DNA sequences. Lately, a new class of compact Cas12 family nucleases, including Cas12f (also known as Cas14), TnpB, and Un1Cas12f1, has emerged as promising candidates for genome editing applications due to their remarkably small size [83,84,85].

Traditional CRISPR nucleases like Cas9 (~1400 amino acids) and Cas12a (~1300 amino acids) are relatively large proteins, which can pose challenges for delivery into cells, especially when using viral vectors with limited cargo capacity. Whereas the small size of these nucleases offers several benefits. Their compact size allows for the inclusion of multiple nucleases or additional regulatory elements within a single delivery vector, enabling complex genetic modifications. In addition, smaller proteins may reduce the likelihood of immune detection, potentially improving safety in therapeutic contexts. While promising, compact Cas12 nucleases currently face challenges such as lower editing efficiency and specificity compared to larger nucleases [85].

Further structural studies and high-throughput screening may aid in the development of improved variants.

dCas9 binds to target DNA without inducing double-strand breaks, allowing it to act as a scaffold for recruiting transcriptional activators or repressors. By fusing dCas9 to activator domains (e.g., VP64 or p300) or repressor domains (e.g., KRAB), researchers can enhance or silence the expression of specific genes in a controlled manner. This approach has been demonstrated to precisely modulate gene expression and phenotypes in mammalian systems, presenting an advantage for studies where gene knockout is either unnecessary or disruptive to the study’s objectives [86,87].

In agricultural research, dCas9-based repression or activation offers a non-permanent way to explore gene function, which may be particularly useful for studying complex traits in livestock.

**Figure 2:**
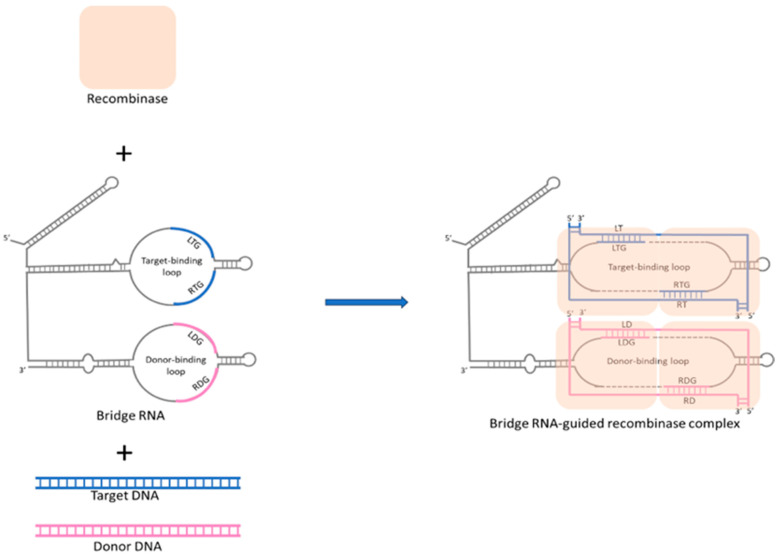
Mechanism of bridge RNA-mediated DNA alignment and recombination in precision genomic editing. Bridge RNA features two independently programmable internal loops: the target-binding loop and the donor-binding loop. The recombinase forms a complex with bridge RNA, then interacts with the target and donor DNA. The target-binding loop (LTG and RTG) pairs with the top and bottom strands of the target DNA, with LTG interacting with the left bottom strand (LT) and RTG interacting with the right top strand (RT) of the target DNA. The donor-binding loop (LDG and RDG) pairs with the top and bottom strands of the donor DNA, with LDG interacting with the left bottom strand (LD) and RDG interacting with the right top strand (RD) of the donor DNA. Through the two binding loops of bridge RNA, donor DNA and target DNA are accurately aligned and catalyzed for recombination, enabling precise genomic editing.

Recently, in 2024, a novel RNA-directed editing methodology, termed bridge RNA-guided recombination systems, was characterized in Escherichia coli [20,88]. This system comprises a recombinase protein that interacts with a guide RNA, akin to the CRISPR-Cas9 mechanism, and the guide RNA specifies two DNA sequences: one for the genomic target site to be altered and the other for the DNA site to be changed. This system utilizes dual-binding loops to facilitate highly specific DNA recombination without introducing double-strand breaks, thus potentially minimizing mutagenic risks. Mechanistically, the bridge RNA confers modular specificity to recombinase enzymes for targeted DNA by using dual-binding loops that recognize and bind to specific sequences in both target and donor DNA, thereby directing precise DNA recombination events, as shown in Figure 2. Excitingly, recent research also confirmed that target-binding and donor-binding loops can be independently reprogrammed to direct sequence-specific recombination between two DNA molecules, which means that bridge RNA-guided recombination systems may offer a unified system for complex genome editing such as precise insertion, excision, and inversion of DNA sequences [20]. Therefore, this system has the potential to broaden the scope and variety of nucleic acid-guided therapeutic approaches beyond CRISPR. Although the efficiency and safety of this system in more complex biological systems remain unclear, the ongoing further research from in vivo studies in eukaryotic cells will pave the way for transforming into an efficient tool for gene editing. Thus, due to its unique advantages compared to CRISPR/Cas9, the bridge RNA-guided recombination system is expected to become the next milestone in the field of gene editing.

## 7. Conclusions

The dual-purpose design of gene-edited pigs combines agricultural productivity enhancements with medical applications, advancing research in organ transplantation and disease modeling. By fulfilling these roles, dual-purpose pigs could reduce costs for producers and offer improved products for consumers. Additionally, the medical applications, particularly in xenotransplantation, hold promise for direct contributions to human health. Recent advancements in genome editing technologies have increased the accessibility of genetically modified pig models for agricultural and biomedical applications. In particular, the effectiveness of the CRISPR/Cas9 system across multiple fields, such as enhancing pigs’ productive performance, viral resistance, generating disease models, and advancing xenotransplantation research, is clearly evident. However, despite its vast potential, the CRISPR/Cas9 system faces several challenges, including off-target effects, DSBs, cellular toxicity, complex edit limitations, multiplexing challenges, and variable targeting efficiency. Fortunately, with the continuous in-depth understanding of DNA cutting and repair mechanisms, numerous efficient gene editing tools continue to emerge. For example, bridge RNA-guided recombination systems position it as a highly promising tool for gene editing applications. In addition, the improved delivery systems, such as lipid nanoparticles and viral vectors, will also facilitate targeting efficiently. It is noteworthy that artificial intelligence is also aiding next-generation sequencing data analysis. The integration of artificial intelligence and machine learning in design processes will also enable the identification of optimal guide RNAs and predict outcomes more effectively [89]. Overall, the continued refinement and application of gene editing technologies will open new avenues for achieving the goals of dual-purpose pigs for both medical and food applications.

## Figures and Tables

**Table 1:** Comparison of CRISPR/Cas9 introduction methods for generating genetically modified pigs.

Introduction Method	Requires Expensive Equipment	Blastocyst Development Rate (%)	Presence of Mosaicism	Reference Number
SCNT	Yes (requires micromanipulation and electroporator)	Moderate (12–26%)	No	[34]
Microinjection	Yes (requires micromanipulation equipment)	High (19–60%)	Yes	[39]
Electroporation	Yes (requires electroporator)	Moderate (20–30%)	Yes	[43]
Lipofection	No	Low (9.5–13.8%)	Yes	[44]

**Table 2:** Application of the CRISPR/Cas9 system in gene editing pigs.

Basic Application Direction	Specific Application Target	Gene Targeted	Reference Number
Improving the Productive Performance of Pigs	Increasing muscle mass	*MSTN*	[5,46]
	Increasing muscle mass via MSTN inhibition	*FST*	[48]
	Alternative approach for muscle mass improvement	*FBXO40*	[31]
	Enhancing muscle yield in native Chinese pig breeds	*IGF2*	[6,49]
	Enhancing pork nutritional value with n-3 PUFAs	*fat-1*	[4]
	Enhancing both muscle mass and meat quality	*fat-1 and IGF-1*	[50]
	Improving thermoregulation and reducing fat	*UCP1*	[51]
Improving the Viral Resistance of Pigs	Increasing resistance to PRRS	*CD163*	[7]
	Reducing susceptibility to coronavirus infections	*ANPEP*	[52]
	Enhancing immunity with antimicrobial properties	*PBD-2*	[53]
	Increasing resistance to CSFV and PRV	*RSAD2*	[54]
Improving Generation of Genetically Modified Disease Models	Cancer model by knocking out tumor suppressor	*RUNX3*	[32]
	Diabetes model focusing on pancreatic development	*PDX1*	[55]
	Hemophilia B model	*Factor IX*	[56]
	Huntington’s disease model	*HTT*	[57]
	Immunodeficient model for cancer research	*IL2RG*	[58]
	Phenylketonuria model	*PAH*	[59]
Improving Xenotransplantation Research	Reducing antigenicity in xenotransplantation	*GGTA1*, *CMAH*, *b4GalNT2*	[63,64]
	Eliminating risk of viral transmission in xenotransplantation	*PERV*	[65,66]
